# Altered Tracer Distribution and Clearance in the Extracellular Space of the Substantia Nigra in a Rodent Model of Parkinson's Disease

**DOI:** 10.3389/fnins.2017.00409

**Published:** 2017-07-25

**Authors:** Yuan Fang, Yanchao Dong, Tao Zheng, Dan Du, Jiexia Wen, Dawei Gao, Lanxiang Liu

**Affiliations:** ^1^Department of Magnetic Resonance Imaging, Qinhuangdao Municipal No. 1 Hospital Qinhuangdao, China; ^2^Department of Interventional Therapy, Qinhuangdao Municipal No. 1 Hospital Qinhuangdao, China; ^3^Department of Central Laboratory, Qinhuangdao Municipal No. 1 Hospital Qinhuangdao, China; ^4^Institute of Chemical and Environmental Engineering, Yanshan University Qinhuangdao, China

**Keywords:** brain clearance, drug delivery, extracellular space, Parkinson's disease, substantia nigra

## Abstract

The relationship between extracellular space (ECS) diffusion parameters and brain drug clearance is not well-studied, especially in the context of Parkinson's disease (PD). Therefore, we used a rodent model of PD to explore the distribution and clearance of a magnetic resonance tracer. Forty male Sprague Dawley rats were randomized into four different groups: a PD group, a Madopar group (PD + Madopar treatment), a sham group, and a control group. All rats received an injection of the extracellular tracer gadolinium-diethylene triaminepentacetic acid (Gd-DTPA) directly into the substantia nigra (SN). ECS diffusion parameters including the effective diffusion coefficient (D^*^), clearance coefficient (k'), ratio of the maximum distribution volume of the tracer (Vd-max%), and half-life (t_1/2_) were measured. We found that all parameters were significantly increased in the PD group compared to the other three groups (D^*^: *F* = 5.774, *p* = 0.0025; k': *F* = 20.00, *P* < 0.0001; Vd-max%: *F* = 12.81, *P* < 0.0001; and t_1/2_: *F* = 23.35, *P* < 0.0001). In conclusion, the PD group exhibited a wider distribution and lower clearance of the tracer compared to the other groups. Moreover, k' was more sensitive than D^*^ for monitoring morphological and functional changes in the ECS in a rodent model of PD.

## Introduction

Parkinson's disease (PD) is a common neurodegenerative disorder that is characterized by progressive motor and cognitive impairments. Although, the pathogenesis of PD remains unclear, the loss of dopaminergic neurons in the substantia nigra has been widely recognized as a major cause of PD (Moradian et al., 2017). Some studies have suggested that the intracellular accumulation of specific proteins is responsible for neuronal dysfunction in PD (Rokad et al., 2016; Svetoni et al., 2016; Wang P. et al., 2016), while other work has identified pathogenic genetic mutations and associated extracellular waste accumulation outside of neurons in PD (Liu et al., 2015; Follett et al., 2016; Lohr et al., 2016). Accordingly, disturbances of the brain extracellular space (ECS) have been implicated in PD pathogenesis (Shi et al., 2015; Lei et al., 2016). The most common treatment consist of administering the drugs levodopa and biperiden, which reduced the extent of the disease and the progress of its symptoms. Sustained and safe delivery of dopamine across the blood brain barrier (BBB) was a major hurdle for successful therapy in Parkinson's disease (PD; Wang N. et al., 2016), however, nanoparticles can delivered dopamine into the brain, reduced dopamine autoxidation-mediated toxicity (Gunay et al., 2016). Moreover, the mechanism of PD was so complicated that it was hard to solve the problem by just add drugs. Therefore, researchers focused on the genetic underpinnings of Parkinson's disease (Ma et al., 2013), and searched for some new methods that may affect the neurodegeneration processes in it (Chen et al., 2016). Such as this, stem cell therapy of PD had shown great potential in retarding the loss of dopaminergic neurons and minimizing the behavioral abnormalities (Salama et al., 2017). In addition, there were also some novel therapies for PD had been used in clinical trials, for example, phytotherapy treatment (Strathearn et al., 2014), levodopa/benserazide microspheres (Yang et al., 2012; Xie et al., 2014), erythropoietin (Qi et al., 2014). As an alternative, some research has focused on direct drug delivery to the brain ECS as a method to bypass the BBB (Barua et al., 2014, 2015).

In order to get this information that diffusion properties of brain ECS, some methods [such as real-time iontophoretic (RTI), integrative optical imaging (IOI), and magnetic resonance (MR)] of qualitatively and quantitatively measuring ECS diffusion parameters had been developed. RTI technique can provide the comprehensive knowledge and the diffusion parameters of ECS with good temporal resolution and versatile ability within the distance range of 10–200 microns (Hrabetova and Nicholson, 2007). And IOI technique can be only applied to measure the ECS parameters in cortex, i.e., around 200 microns, since the optical signal in deep brain cannot be received by a charge coupled device (CCD; Thorne et al., 2008). MRI had been widely used for *in vivo* imaging of biological tissue, because of its advantages of relatively low ionization damage, high resolution of soft tissue. So far, MRI was the only imaging method to detect the brain ECS in whole brain scale. And also was the only measurement that provided a 3-D visualization of the dynamic drainage flow of tracer at global view.

Recently, MR imaging tracer-based methods such asgadolinium-diethylene triaminepentacetic acid (Gd-DTPA) have been applied to image the rat brain ECS (Han et al., 2014). Gd-DTPA was selected as a probe on the basis of its biomedical inertness, thermal stability, and relatively small molecular weight; additionally, Gd-DTPA primarily accumulates in the ECS without entering cerebral neurons (Han et al., 2014). The use of Gd-DPTA as a MRI tracer not only allowed the visualization of dynamic brain clearance, but also the comprehensively measured for brain ECS diffusion parameters including the effective diffusion coefficient (D^*^), clearance coefficient (k'), ratio of the maximum distribution volume of the tracer (Vd-max%), and half-life (t_1/2_). In a previous study, this method was used to image the ECS of the substantia nigra (SN) in a rat model of PD (Ablat et al., 2016; Ren et al., 2016). Yet, very few studies have examined the relationship between diffusion parameters and drug clearance from the brain ECS. Therefore, the purpose of this study was to investigate how changes in the properties of the brain ECS relate to pathological changes in PD.

## Materials and methods

### Animals

All experiments were performed on mature (7-weeks-old; 200–250 g) male Sprague-Dawley rats (*n* = 40) and were conducted in accordance with the national guidelines for the use of experimental animals. All experimental protocols were approved by the Ethics Committee of Qinhuangdao Municipal No. 1 Hospital. Rats were housed in a room with controlled temperature (22 ± 2°C) and humidity (60 ± 5%) on a 12 h light/dark cycle with *ad libitum* access to food and water. Rats were randomly divided into four groups: (1) a PD model group (*n* = 10), (2) Madopar group (*n* = 10), (3) sham group (*n* = 10) and (4) control group (*n* = 10).

### Surgical procedure

The procedure for PD model induction was as follows. First, the rat was anesthetized using compound anesthetic by intraperitoneal injection (3 ml/kg) and maintained under anesthesia (~2 ml/kg/h) for the duration of the operation. A heating pad setting to 38 ± 0.5°C was placed under the rat to maintain an appropriate body temperature. The rat was mounted into a stereotactic apparatus (Lab Standard Stereotaxic-Single, Stoelting Co, Illinois, USA), bregma was exposed, an incision was made on the scalp along the sagittal suture, and a small trephine hole was drilled in accordance with the stereotactic coordinates of the right SN (relative to bregma: AP, −4.8 mm; ML, 1.9 mm; DV, −7.8 mm). Using a microsyringe (Hamilton Bonaduz AG, Bonaduz, Switzerland), 6 μl of 6-OHDA solution (2 μg/μl in normal saline containing 0.2% ascorbate; Sigma Chemical Co., St. Louis, MO, USA) was automatically infused into the SN of rats in the PD and Madopar groups or an identical volume of saline containing 0.2% ascorbate was automatically infused into the SN of rats in the sham group at a rate of 1 μl/min. The needle was kept in place for 5 min after completion of the injection and then slowly withdrawn. The scalp was sutured closed and intramuscular antibiotics were administered to prevent infection. Two weeks after the operation, rats in Madopar group began to receive treatment with Madopar (20 mg/kg) twice daily (at 09:00 and 15:00) by oral gavage until MR scanning.

### Rotarod test

PD and Madopar group rats received single intraperitoneal injections of apomorphine (0.5 mg/kg in normal saline) at the beginning of the 1st, 2nd, 4th, and 6th weeks after surgery. For rotarod testing, animals were allowed to habituate to the test apparatus for 10 min and then for an additional 2 min after the injection. Full rotations were counted in a cylindrical container in a dimlylit, quiet room. Rotational asymmetry was scored continuously for 30 min and then complete contralateral rotation times were scored. Rats with test scores >7 were retained for the study and analysis.

### MR scanning

Anesthesia was performed as described above and rats were scanned using a 3.0-T MRI system (Verio, Siemens Medical Solutions, Erlangen, Germany) with an eight-channel coil. Brain images were obtained with a high-resolution T1-weighted 3-dimensional magnetization-prepared rapid-acquisition with gradient echo (T1 3DMP-RAGE) sequence with the parameters specified by Han and Zuo (Han et al., 2014; Zuo et al., 2015). A 2-μl volume of 10 mmol/L Gd-DTPA solution (Magnevist; Bayer Schering Pharma AG, Berlin, Germany) was injected directly into the SN as described above (see Section Surgical Procedure).

### Immunohistochemistry

Brain tissues were prepared as described previously. Briefly, each brain was sliced into 8-μm coronal sections through the ventral mesencephalon. Sections of the SN were selected for immunohistochemistry and matched between samples as closely as possible. Immunohistochemistry for tyrosine hydroxylase (TH) and aquaporin-4 (AQP4) was performed by overnight incubation with the appropriate primary antibody (rabbit anti-rat; 1:300; Abcam, Cambridge, UK) at 4°C. Sections were next incubated with secondary antibody for 30 min at 37°C. Sections were visualized with 3, 3′-diaminobenzidine and nuclei were counterstained with hematoxylin and eosin (HE). Transverse frozen sections (5 μm) were dried and then soaked overnight in a 1:1 mixture of alcohol and chloroform in the dark at 22 ± 1°C. Sections were rehydrated on the following day and stained with 0.1% cresyl violet solution (Sigma, St. Louis, MO, USA) for 5 min. Differentiation, dehydration, and rinsing were performed as described previously. Finally, sections were mounted with Permount (Beyotime Institute of Biotechnology, Shanghai, China) and observed under a light microscope (Olympus) equipped with a CCD camera (Leica DMI4000B, Germany). Surviving neurons were counted using Photoshop CS3 software (Adobe).

### Statistical analysis

Statistical analyses were conducted using SPSS statistical software, version 21.0(IBM SPSS Statistics for Windows, Armonk, New York, USA). Data (D^*^, k', Vd-max% and t_1/2_) are presented as the mean ± standard deviation. Significant between-group differences were evaluated using a 1-way analysis of variance (ANOVA) and least significant difference (LSD) *post-hoc* tests. Pearson correlation analyses were employed to assess relationships among D^*^, k', Vd-max%, and t_1/2_ in the PD group. *P* < 0.05 was used as the threshold for statistical significance.

## Results

### Comparison of ECS diffusion parameters

**Figure 1:**
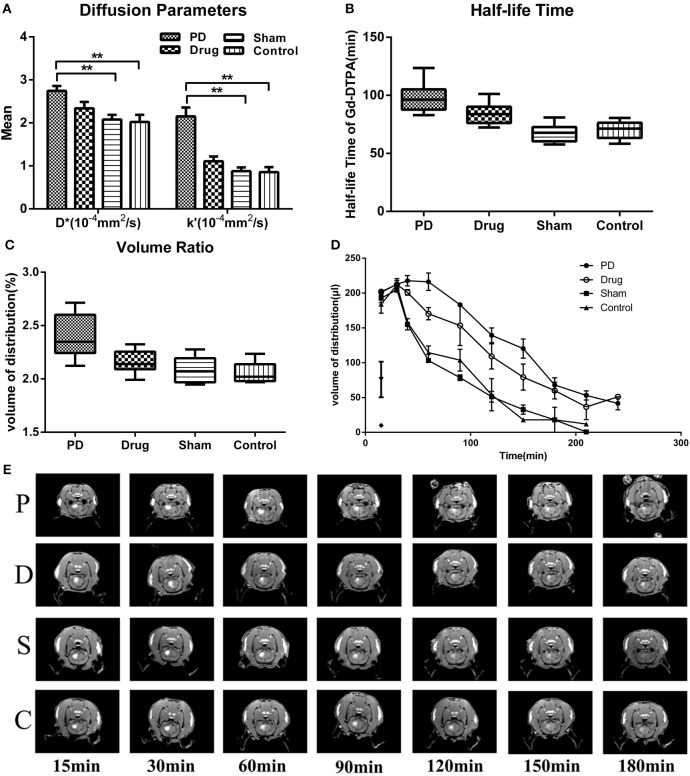
Statistical analysis of diffusion parameters between groups. **(A)** Comparison of D^*^ and k'-values. **(B)** Comparison of Vd-max% values. **(C)** Comparison of t_1/2_ values. **(D)** Gd-DTPA volume of distribution ratio-time curves. **(E)** Coronal images after Gd-DTPA injection. Images were selected according to maximum range of Gd-DTPA. Window Level (WL): 2000, Window Width (WW): 3000. Control, control group; Drug, Madopar-treated Parkinson's disease model group; PD, Parkinson's disease model group; Sham, sham group. ^**^P < 0.001, ^*^P < 0.001.

We found that D^*^was higher in the PD group than that of the other three groups ([2.744 ± 0.341] × 10^−4^ mm^2^/s in the PD group vs. [2.340 ± 0.448] × 10^−4^ mm^2^/s in the Madopar drug group, [2.078 ± 0.326] × 10^−4^ mm^2^/s in the sham group, and [2.023 ± 0.501] × 10^−4^ mm^2^/s in the control group; *F* = 5.774, *P* = 0.0025, Figure 1A); differences among the Madopar, sham, and control groups were non-significant (*P* = 0.185 and *P* = 0.111, respectively; LSD). The k' parameter was also higher in the PD group than that of the other three groups ([2.153 ± 0.610] × 10^−4^ mm^2^/s in the PD group vs. [1.109 ± 0.333] × 10^−4^ mm^2^/s in the Madopar group, [0.879 ± 0.262] × 10^−4^ mm^2^/s in the sham group, and [0.854 ± 0.355] × 10^−4^ mm^2^/s in the control group; *F* = 20.00, *P* < 0.0001, Figure 1A). Distribution volume-time profiles are shown in Figure 1D and demonstrate that the Vd-max% of Gd-DTPA was higher in the PD group (2.392 ± 0.185%) than that of the Madopar group (2.153 ± 0.102%), sham group (2.091 ± 0.110%), and control group (2.054 ± 0.090%; *F* = 12.81, *P* < 0.0001, Figure 1B). The value of t_1/2_ was also significantly higher in the PD group (97.839 ± 11.874 min) than that of the other three groups (84.084 ± 8.157 min in the Madopar group, 67.374 ± 7.222 min in the sham group, and 69.649 ± 7.017 min in the control group; *F* = 23.35, *P* < 0.0001, Figure 1C).

### Relationships among diffusion parameters

**Figure 2:**
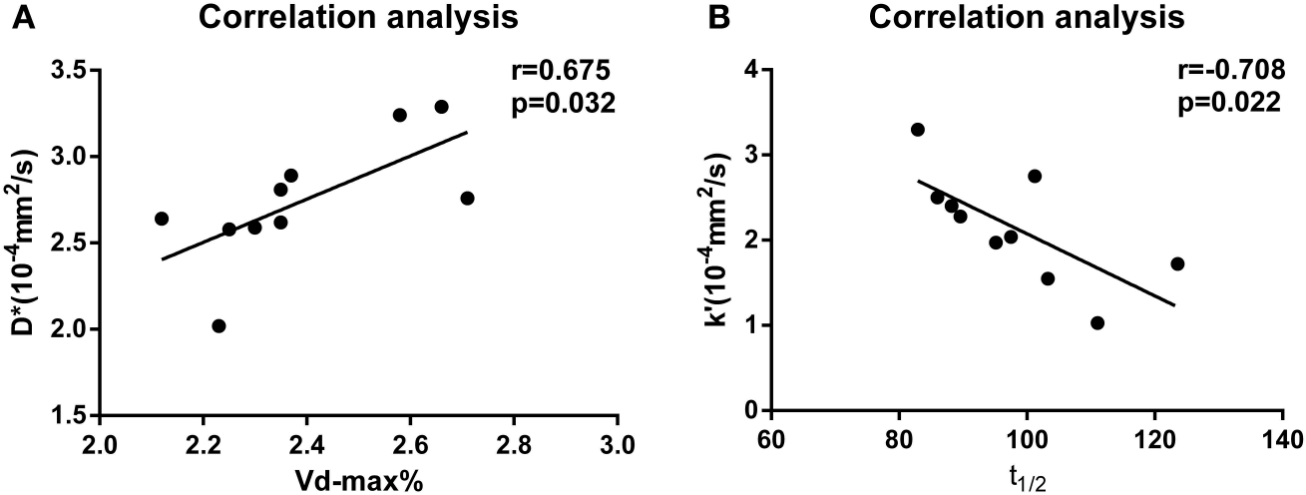
**(A)** Correlation analysis of D^*^ and Vd-max% values for the PD group. **(B)** Correlation analysis of k' and t_1/2_ values for the PD group.

In the PD group, there was a positive correlation between D^*^ and Vd-max% (*P* = 0.032, *r* = 0.675, Figure 2A). In contrast, there was a significant negative correlation between k' and t_1/2_ in the PD group (*P* = 0.022, *r* = −0.708, Figure 2B).

### Comparison of TH+ and AQP4+ neurons

**Figure 3:**
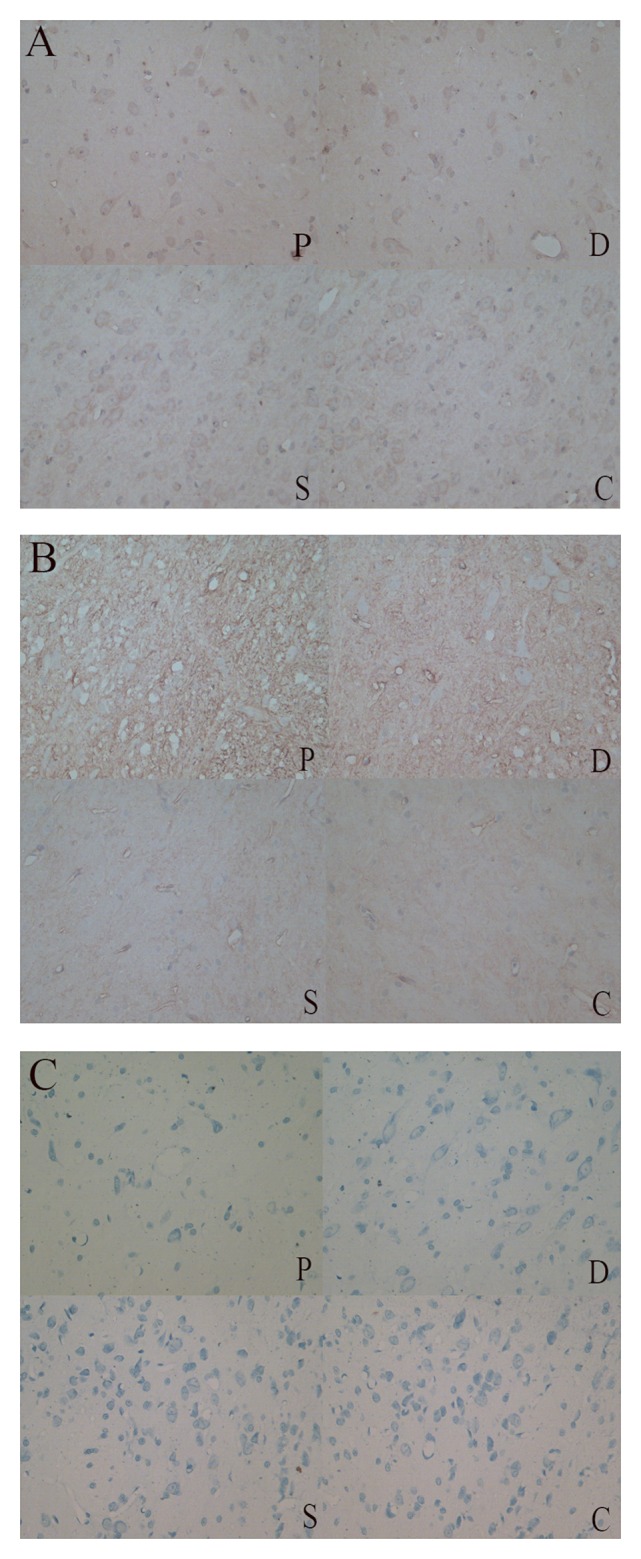
**(A)** TH+ neurons were more frequently and easily detectable in the sham and control group SNs compared to the PD and Madobar group. **(B)** AQP4+ area was markedly increased in the PD and Madopar group SN compared to the sham group and control group. AQP4 expression in the Madopar group was lower than that observed in the PD group. **(C)** Neuronal degeneration and neuronal loss were greater with larger observable damaged area in the PD group than that of the other three groups.

Rats that received 6-OHDA injections exhibited obvious apoptosis of TH+ cells in the SN (Figure 3A). The extent of neuronal loss in the Madopar group was qualitatively less than that observed in the PD group. TH+ neurons were more frequently and easily detectable in the sham group and control group SNs compared to the other two groups. Additionally, AQP4+ area was markedly increased in the 6-OHDA-lesioned SN compared to the other groups. AQP4 expression in the Madopar group was lower than that observed in the PD group. Moreover, there were fewer AQP4+ positive neuronsin the PD group than that of the Madopar group (Figure 3B). Nissl staining revealed that neuronal degeneration and neuronal loss were greater with larger observable damaged area in the PD group than that of the other three groups (Figure 3C).

## Discussion

### Diffusion parameters of PD rats

It is widely believed that the brain ECS is a highly interconnected system that exhibits regional heterogeneity in various diffusion parameters (Li et al., 2015). Though some studies have examined the SN ECS in normal rats, this is one of the first investigations to examine the SN ECS in a rodent model of PD. Using an MRI tracer-based method, we found that D^*^ and k'-values, which respectively characterize local ECS diffusion and clearance on a microscopic level, were higher in the 6-OHDA-lesioned SN than that of the non-lesioned SN. Moreover, Vd-max% and t_1/2_, which reflected drug diffusion and clearance on a macroscopic level, showed similar patterns of elevation in the PD group. One possible reason for our findings related to the observed apoptosis of dopaminergic neurons and previously reported glial proliferation in PD model rats. A previous study indicated that the volume of glial cells was significantly less than that of dopaminergic neurons in the SN of PD model rats, suggesting that changes in D^*^-values are mainly related to the number of dopaminergic neurons (Ren et al., 2016). In our study, D^*^-values were lower in the Madopar group than that of the PD group, but were not significantly different between the Madopar group and the sham or control groups. It can be inferred that Madopar treatment partially preserved dopaminergic neurons, which was consistent with previous work (Pan et al., 2015) as well as other findings in our study. Yet, our observation of increased k'-values in the PD group compared to other groups was inconsistent with previous research reporting reduced k'-values in the SN of PD model rats; it was proposed that glial cell hyperplasia in the SN area and waste accumulation (e.g., reduced α-synclearance) in the SN was responsible for decreased k'-values (Ren et al., 2016). In our study, we speculated that the overexpression of AQP4 on proliferating glial cell membranes was responsible for observed increases in k' (Ikeshima-Kataoka, 2016; Tham et al., 2016). AQP4 in the brain ECS positively influenced the transport rate of water molecules (Liu et al., 2016; Vindedal et al., 2016). To this end, some researches have shown that AQP4 overexpression increases ECS clearance coefficient values in the brain (Zuo et al., 2015). It was possible that the reason for decreased k'-values in the Madopar group compared to the PD group but not the other groups was due to a suppressive effect of treatment on glial cell hyperplasia and AQP4 expression.

Interestingly, we found that k'-values in the PD group were increased to a greater extent than D^*^-values. We speculated that this was related to glial oxidative stress and hyperplasia occurring in the early stage of the PD model, which initially produced dopaminergic neuroprotection and thereby prevented the overt depletion of dopaminergic neurons. As mentioned above, changes in D^*^-value were mainly related to the number of dopaminergic neurons (Garbayo et al., 2016). Therefore, a reasonable hypothesis was that k'-values may be more sensitive than D^*^-values for monitoring the brain ECS. To this end, k'-values may provide better utility and sensitivity for evaluating the morphology and function of the ECS in the SN of PD model rats.

### Distribution and clearance of drugs

Vd-max% estimates the maximum distribution volume of a given drug in the brain ECS. We found that Vd-max% of the SN was higher in the PD group than that of the other three groups, indicating that harmful substances such as α-syn were retained longer and distributed more widely in the PD model SN, increasing the likelihood of toxicity (Han et al., 2014; Ren et al., 2016). A previous study investigated tracer clearance in the normal rat SN ECS, but did not clearly establish clearance timesor diffusion ranges for the tracer (Han et al., 2014). Our study found that t_1/2_ values were increased in the PD group compared to the other three groups, consistent with the above finding regarding Vd-max%. Several potential rationales can explain these observations. First, the accumulation of waste in the brain ECS may have competed for tracer clearance. Previous research has demonstrated that neurons in PD model rats produce abnormally large amounts of waste (e.g., α-syn; Stuendl et al., 2016; Kim et al., 2017). Therefore, metabolite accumulation in the brain ECS of PD rats may increase tracer t_1/2_ values. Second, other studies have reported effects of local neuronal excitability on the brain ECS, including in models of PD. Shi et al. reported that the rate of water molecule diffusion was decreased in the ECS of excited brain areas (Shi et al., 2015). Indeed, t_1/2_ values were affected by neural activity during the sleep and wake states (Xie et al., 2013). By this logic, decreased neuronal excitability in PD did not favor the brain clearance of harmful substances. Consistent with this hypothesis, t_1/2_ was decreased in the Madopar group compared to the PD group, but was still higher in the Madopar group than that of the sham group.

Finally, we observed a positive correlation between Vd-max% and D^*^in the SN of PD model rats. Increased D^*^-values in the PD group were likely due to the loss of dopaminergic neurons, leading to decreased tortuosity of the brain ECS. Yet, there was no correlation between Vd-max% and k' in other groups. Moreover, t_1/2_ was negatively correlated with k' in the PD group. If the rate of water molecule diffusion was higher, it can be expected that the value of t_1/2_ would be smaller. Given that Vd-max% and t_1/2_ values reflected a wider distribution and lower clearance rate of tracer in the ECS of PD group animals, we speculated that Vd-max% was mainly affected by D^*^, and t_1/2_ was mainly affected by k'.

## Conclusion

In conclusion, Vd-max% and t_1/2_ values reflected a wider distribution and lower clearance rate in the SN ECS of PD model rats. Additionally, we speculated that k' was more sensitive than D^*^ for monitoring morphological and functional changes in the ECS of the rat SN. These findings have important implications for the treatment of PD via the direct application of therapeutics to the brain ECS as a new approach for drug delivery.

## Author contributions

LL and YF designed experiments; YD, TZ, and DD carried out experiments; JW analyzed experimental results. YD analyzed sequencing data and developed analysis tools. DG assisted with Illumina sequencing. LL, YD, and TZ wrote the manuscript.

### Conflict of interest statement

The authors declare that the research was conducted in the absence of any commercial or financial relationships that could be construed as a potential conflict of interest.

## References

[B1] Ablat N. Lv D. Ren R. Xiaokaiti Y. Ma X. Zhao X.. (2016). Neuroprotective effects of a standardized flavonoid extract from safflower against a rotenone-induced rat model of Parkinson's Disease. Molecules 21:E1107. 10.3390/molecules21091107 27563865 PMC6274364

[B2] Barua N. U. Bienemann A. S. Woolley M. Wyatt M. J. Johnson D. Lewis O.. (2015). Convection-enhanced delivery of MANF–volume of distribution analysis in porcine putamen and substantia nigra. J. Neurol. Sci. 357, 264–269. 10.1016/j.jns.2015.08.003 26276514

[B3] Barua N. U. Gill S. S. Love S. (2014). Convection-enhanced drug delivery to the brain: therapeutic potential and neuropathological considerations. Brain Pathol. 24, 117–127. 10.1111/bpa.12082 23944716 PMC8028869

[B4] Chen W. Li H. Liu Z. Yuan W. (2016). Lipopolyplex for therapeutic gene delivery and its application for the treatment of Parkinson's Disease. Front. Aging Neurosci. 8:68. 10.3389/fnagi.2016.00068 27092073 PMC4820442

[B5] Follett J. Bugarcic A. Yang Z. Ariotti N. Norwood S. J. Collins B. M.. (2016). Parkinson disease-linked Vps35 R524W mutation impairs the endosomal association of retromer and induces alpha-synuclein aggregation. J. Biol. Chem. 291, 18283–18298. 10.1074/jbc.M115.703157 27385586 PMC5000076

[B6] Garbayo E. Ansorena E. Lana H. Carmona-Abellan M. D. Marcilla I. Lanciego J. L.. (2016). Brain delivery of microencapsulated GDNF induces functional and structural recovery in parkinsonian monkeys. Biomaterials 110, 11–23. 10.1016/j.biomaterials.2016.09.015 27697668

[B7] Gunay M. S. Ozer A. Y. Chalon S. (2016). Drug delivery systems for imaging and therapy of Parkinson's Disease. Curr. Neuropharmacol. 14, 376–391. 10.2174/1570159X14666151230124904 26714584 PMC4876593

[B8] Han H. Shi C. Fu Y. Zuo L. Lee K. He Q.. (2014). A novel MRI tracer-based method for measuring water diffusion in the extracellular space of the rat brain. IEEE J. Biomed. Health Informatics 18, 978–983. 10.1109/JBHI.2014.2308279 24808229

[B9] Hrabetova S. Nicholson C. (2007). Chapter 10: Biophysical properties of brain extracellular space explored with ion-selective microelectrodes, integrative optical imaging and related techniques, in Electrochemical Methods for Neuroscience, eds Michael A. C. Borland L. M. (Boca Raton, FL: Taylor & Francis Group, LLC). 21204394

[B10] Ikeshima-Kataoka H. (2016). Neuroimmunological implications of AQP4 in astrocytes. Int. J. Mol. Sci. 17:1306. 10.3390/ijms17081306 27517922 PMC5000703

[B11] Kim J.-S. Park I.-S. Park H.-E. Kim S.-Y. Yun J. A. Jung C. K.. (2017). α-Synuclein in the colon and premotor markers of Parkinson disease in neurologically normal subjects. Neurol. Sci. 38, 171–179. 10.1007/s10072-016-2745-0 27803984

[B12] Lei Y. Han H. Yuan F. Javeed A. Zhao Y. (2016). The brain interstitial system: anatomy, modeling, *in vivo* measurement, and applications. Prog. Neurobiol. [Epub ahead of print]. 10.1016/j.pneurobio.2015.12.007 26837044

[B13] Li H. Y. Zhao Y. Zuo L. Fu Y. Li N. Yuan L.. (2015). Diffusion of fluorescent and magnetic molecular probes in brain interstitial space. Beijing Da Xue Xue Bao 47, 667–673. 26284407

[B14] Liu C. Cholerton B. Shi M. Ginghina C. Cain K. C. Auinger P.. (2015). CSF tau and tau/Abeta42 predict cognitive decline in Parkinson's disease. Parkinson. Relat. Disord. 21, 271–276. 10.1016/j.parkreldis.2014.12.027 25596881 PMC4603566

[B15] Liu S. Mao J. Wang T. Fu X. (2016). Downregulation of aquaporin-4 protects brain against hypoxia ischemia via anti-inflammatory mechanism. Mol. Neurobiol. [Epub ahead of print]. 10.1007/s12035-016-0185-8 27726111

[B16] Lohr K. M. Masoud S. T. Salahpour A. Miller G. W. (2016). Membrane transporters as mediators of synaptic dopamine dynamics: implications for disease. Eur. J. Neurosci. 45, 20–33. 10.1111/ejn.13357 27520881 PMC5209277

[B17] Ma L. Wei L. Wu F. Hu Z. Liu Z. Yuan W. (2013). Advances with microRNAs in Parkinson's disease research. Drug Des. Dev. Ther. 7, 1103–1113. 10.2147/DDDT.S48500 24109179 PMC3792848

[B18] Moradian H. Keshvari H. Fasehee H. Dinarvand R. Faghihi S. (2017). Combining NT3-overexpressing MSCs and PLGA microcarriers for brain tissue engineering: a potential tool for treatment of Parkinson's disease. Mater. Sci. Eng. C 76, 934–943. 10.1016/j.msec.2017.02.178 28482609

[B19] Pan X. Chen C. Huang J. Wei H. Fan Q. (2015). Neuroprotective effect of combined therapy with hyperbaric oxygen and madopar on 6-hydroxydopamine-induced Parkinson's disease in rats. Neurosci. Lett. 600, 220–225. 10.1016/j.neulet.2015.06.030 26101828

[B20] Qi C. Xu M. Gan J. Yang X. Wu N. Song L.. (2014). Erythropoietin improves neurobehavior by reducing dopaminergic neuron loss in a 6hydroxydopamineinduced rat model. Int. J. Mol. Med. 34, 440–450. 10.3892/ijmm.2014.1810 24939444 PMC4094589

[B21] Ren R. Shi C. Cao J. Sun Y. Zhao X. Guo Y.. (2016). Neuroprotective effects of A standardized flavonoid extract of safflower against neurotoxin-induced cellular and animal models of Parkinson's Disease. Sci. Rep. 6:22135. 10.1038/srep22135 26906725 PMC4764910

[B22] Rokad D. Ghaisas S. Harischandra D. S. Jin H. Anantharam V. Kanthasamy A.. (2016). Role of neurotoxicants and traumatic brain injury in α-synuclein protein misfolding and aggregation. Brain Res. Bull. [Epub ahead of print]. 10.1016/j.brainresbull.2016.12.003 27993598 PMC5623095

[B23] Salama M. Sobh M. Emam M. Abdalla A. Sabry D. El-Gamal M.. (2017). Effect of intranasal stem cell administration on the nigrostriatal system in a mouse model of Parkinson's disease. Exp. Ther. Med. 13, 976–982. 10.3892/etm.2017.4073 28450929 PMC5403256

[B24] Shi C. Lei Y. Han H. Zuo L. Yan J. He Q.. (2015). Transportation in the interstitial space of the brain can be regulated by neuronal excitation. Sci. Rep. 5:17673. 10.1038/srep17673 26631412 PMC4668547

[B25] Strathearn K. E. Yousef G. G. Grace M. H. Roy S. L. Tambe M. A. Ferruzzi M. G.. (2014). Neuroprotective effects of anthocyanin- and proanthocyanidin-rich extracts in cellular models of Parkinsons disease. Brain Res. 1555, 60–77. 10.1016/j.brainres.2014.01.047 24502982 PMC4024464

[B26] Stuendl A. Kunadt M. Kruse N. Bartels C. Moebius W. Danzer K. M.. (2016). Induction of alpha-synuclein aggregate formation by CSF exosomes from patients with Parkinson's disease and dementia with Lewy bodies. Brain 139, 481–494. 10.1093/brain/awv346 26647156 PMC4805087

[B27] Svetoni F. Frisone P. Paronetto M. P. (2016). Role of FET proteins in neurodegenerative disorders. RNA Biol. 13, 1089–1102. 10.1080/15476286.2016.1211225 27415968 PMC5100351

[B28] Tham D. K. Joshi B. Moukhles H. (2016). Aquaporin-4 cell-surface expression and turnover are regulated by dystroglycan, dynamin, and the extracellular matrix in astrocytes. PLoS ONE 11:e0165439. 10.1371/journal.pone.0165439 27788222 PMC5082936

[B29] Thorne R. G. Lakkaraju A. Rodriguez-Boulan E. Nicholson C. (2008). *In vivo* diffusion of lactoferrin in brain extracellular space is regulated by interactions with heparan sulfate. Proc. Natl. Acad. Sci. U.S.A. 105, 8416–8421. 10.1073/pnas.0711345105 18541909 PMC2448851

[B30] Vindedal G. F. Thoren A. E. Jensen V. Klungland A. Zhang Y. Holtzman M. J.. (2016). Removal of aquaporin-4 from glial and ependymal membranes causes brain water accumulation. Mol. Cell. Neurosci. 77, 47–52. 10.1016/j.mcn.2016.10.004 27751903 PMC5157926

[B31] Wang N. Jin X. Guo D. Tong G. Zhu X. (2016). Iron chelation nanoparticles with delayed saturation as an effective therapy for Parkinson Disease. Biomacromolecules 18, 461–474. 10.1021/acs.biomac.6b01547 27989126

[B32] Wang P. Li X. Li X. Yang W. Yu S. (2016). Blood plasma of patients with Parkinson's disease increases alpha-synuclein aggregation and neurotoxicity. Parkinsons Dis. 2016:7596482. 10.1155/2016/7596482 27965913 PMC5124690

[B33] Xie C. L. Wang W. W. Zhang S. F. Yuan M. L. Che J. Y. Gan J.. (2014). Levodopa/benserazide microsphere (LBM) prevents L-dopa induced dyskinesia by inactivation of the DR1/PKA/P-tau pathway in 6-OHDA-lesioned Parkinson's rats. Sci. Rep. 4:7506. 10.1038/srep07506 25511986 PMC4267205

[B34] Xie L. Kang H. Xu Q. Chen M. J. Liao Y. Thiyagarajan M.. (2013). Sleep drives metabolite clearance from the adult brain. Science 342, 373–377. 10.1126/science.1241224 24136970 PMC3880190

[B35] Yang X. Chen Y. Hong X. Wu N. Song L. Yuan W.. (2012). Levodopa/benserazide microspheres reduced levodopa-induced dyskinesia by downregulating phosphorylated GluR1 expression in 6-OHDA-lesioned rats. Drug Des. Devel. Ther. 6, 341–347. 10.2147/DDDT.S38008 23185117 PMC3506046

[B36] Zuo L. Li K. Han H. (2015). Comparative analysis by magnetic resonance imaging of extracellular space diffusion and interstitial fluid flow in the rat striatum and thalamus. Appl. Magn. Reson. 46, 623–632. 10.1007/s00723-015-0670-7

